# Levels and Modifications of Both Lens Fiber Cell Connexins Are Affected in Connexin Mutant Mice

**DOI:** 10.3390/cells11182786

**Published:** 2022-09-07

**Authors:** Oscar Jara, Peter J. Minogue, Viviana M. Berthoud, Eric C. Beyer

**Affiliations:** Department of Pediatrics, University of Chicago, Chicago, IL 60637, USA

**Keywords:** connexin, cataracts, lens, connexin50, connexin46

## Abstract

In the lens, cell homeostasis and transparency are supported by intercellular communication facilitated by the channels formed of connexin46 (Cx46) and connexin50 (Cx50). Mutations of these connexins are linked to inherited cataracts. We studied the levels and the variations in electrophoretic mobilities of the immunoreactive Cx46 and Cx50 bands between 1 and 21 days after birth in the lenses of wild-type mice and homozygous animals from two different mouse models of connexin-linked cataracts (Cx46fs380 and Cx50D47A). In Cx50D47A mice, the expression of the mutant Cx50 reduced the normal phosphorylation of the co-expressed wild-type Cx46. In both models, levels of the mutant connexin and the co-expressed wild-type connexin decayed more rapidly than in wild-type mice but with different time courses. In the Cx46fs380 mice, modeling suggested that Cx50 degradation could be explained by the mixing of mutant Cx46 with wild-type Cx50. However, in Cx50D47A mice, similar modeling suggested that mixing alone could not explain the decrease in Cx46 levels. These data highlight the complex influences between two connexin proteins expressed in the same cell, some of which occur through direct mixing, while others occur indirectly, as in Cx50D47A mice, where the expression of the mutant connexin causes endoplasmic reticulum stress and impaired differentiation.

## 1. Introduction

Communication between cells is a critical process in all tissues within the body. Direct intercellular communication is facilitated by the passage of ions and small molecules through the channels contained in gap junctions. A gap junction channel is formed by the docking of two hemichannels (one in the plasma membrane of each of the apposing cells) that each contain six subunit proteins called connexins (reviewed in [1]). The connexins form a closely related family with 21 different members expressed in humans and 20 in mice [2,3]. Although an intercellular channel can be formed by one connexin subtype, many cells express more than one connexin subtype, allowing for the formation of channels containing combinations of different connexin subtypes.

The lens is a transparent and avascular organ formed by epithelial and fiber cells. During development, epithelial cells in the equatorial region differentiate into fiber cells, a process that involves cell elongation and the loss of the organelles. Gap junctions and the intercellular communication that they support are critical for the survival of the lens and the maintenance of transparency because they are key components of the internal circulation of water and ions that supports lens homeostasis [4]. While most studies have focused on the importance of gap junctions for normal physiology and the development of disease, the lens is also an excellent system to study the cell biology and biochemistry of the connexin proteins under normal (physiological) and pathological conditions. The fiber cells that comprise the bulk of the lens predominantly express two connexins, connexin46 (Cx46) and connexin50 (Cx50). At least some of the gap junction hemichannels contain both connexins [5,6]. Therefore, the lens is also an excellent system with which to study interactions between co-expressed connexins.

Mutations in the genes encoding Cx46 and Cx50 cause cataracts [7]. We have been studying two mouse models that mimic human congenital cataracts, one expressing a frameshift mutation in Cx46 (Cx46fs380) [8] and the other expressing a missense mutation in Cx50 (Cx50D47A) [9]. In both models, gap junction-mediated fiber cell coupling is reduced (when studied at 2–3 months of age) and cataracts develop through calcium accumulation and precipitation [10,11,12]. However, the timing of cataract development is different between these mouse models. In both homozygous and heterozygous Cx50D47A mice, lens opacities are detectable at 1 month of age. In contrast, in Cx46fs380 mice, opacities are not observed until about 2 months in homozygotes and 4 months (or later) in heterozygotes. At any given age, the cataracts are more severe in Cx50D47A mice than in Cx46fs380 mice. While the sizes of the lenses are similar in wild-type and Cx46fs380 mice, differentiation and growth are impaired in Cx50D47A mutant lenses. Interestingly, even though only one connexin carries a mutation, the levels of both Cx46 and Cx50 are severely reduced in heterozygous and homozygous of both mouse line [8,9,10,12].

Most of our previous quantitative studies of connexin abundance in lenses of the connexin mutant mice were performed on mice that were at least one month old. The current study was designed to evaluate the time-course of changes in Cx46 and Cx50 abundance at earlier post-natal ages in wild-type mice and in homozygotes from these two mutant mouse lines. We have also examined how the expression of a mutant connexin in the lens affects the post-translational modifications of the co-expressed wild-type connexin (as detected by changes in electrophoretic mobility patterns).

## 2. Materials and Methods

### 2.1. Mice

Wild-type and homozygous Cx50D47A mutant mice (also known as *No2* or ENU-326) [13] were maintained in the C3H mouse strain. Wild-type and homozygous Cx46fs380 mice were maintained on a mixed 129/C57BL6 background, as previously described [8]. All experiments were performed using animals that were wild-type or homozygous for the mutant connexin. All animal procedures were performed according to protocols approved by the University of Chicago Animal Care and Use Committee.

### 2.2. Immunoblotting

Lenses were harvested at 1, 4, 7, 14, and 21 days after birth in phosphate-buffered saline pH 7.4 (PBS). After dissection, lenses were homogenized in PBS containing 20 mM NaF, 10 mM Na_3_VO_4_, 2 mM phenylmethylsulfonyl fluoride (PMSF), 4 mM EDTA, and 1 tablet/7 mL cOmplete EDTA-free protease inhibitor cocktail (Roche Applied Science, Indianapolis, IN, USA), using a glass–glass homogenizer, followed by sonication. Homogenates were kept at −80 °C until use. The concentration of proteins in the homogenates was determined using the BioRad Protein Assay (BioRad, Hercules, CA, USA), a method based on the Bradford dye-binding procedure [14].

Immunoblots were performed as previously described [15,16,17,18]. Homogenate aliquots containing equal amounts of protein (100 µg for detection of Cx46 and 30 µg for detection of Cx50) were loaded in each lane. Proteins were resolved in sodium dodecyl sulfate-containing 8% polyacrylamide gels. After electrotransfer of the proteins onto Immobilon P membranes (Millipore, Bedford, MA, USA) using a wet transfer apparatus, membranes were stained with Ponceau S to confirm equivalence of loading and transfer, as previously performed [15]. Membranes were blocked with 5% non-fat dry milk in Tris buffered saline, pH 7.4, and then incubated with previously described affinity purified anti-Cx50 antibodies (directed against the carboxyl terminus of Cx50 (amino acids 231–433) [17] or previously described anti-Cx46 antibodies (directed against a synthetic peptide sequence from within the intracellular loop of mouse Cx46 (amino acids 109–129) [9] overnight at 4 °C. Membranes were rinsed and incubated with peroxidase-conjugated goat anti-rabbit IgG antibodies (catalog number: 111-035-144; lot number: 157038; Jackson ImmunoResearch Laboratories, Inc., West Grove, PA, USA) for 1 hour at room temperature. The binding of secondary antibodies was detected using enhanced chemiluminescence GE Healthcare Amersham ECL Western Blotting Detection Reagents (ThermoFisher Scientific, Waltham, MA, USA).

The intensity of the bands was analyzed by densitometry using Adobe Photoshop (Adobe Systems, Inc., San Jose, CA, USA). Results are reported in arbitrary units that represent the density of the immunoreactive connexin bands. Comparisons are facilitated by loading a constant amount of lens protein in each lane. We have previously shown that this is more accurate for the assessment of changes in lens proteins than normalization to a “housekeeping” protein since there are substantial variations in the levels of those proteins with lens development, aging, and disease [15]. Three sets of independent experiments containing all ages were performed. Statistical significance was assessed using ANOVA; *p* < 0.05 was considered significant.

All studies were performed by comparing connexin levels in wild-type and mutant mice of the same line to avoid any confounding effects related to genetic differences. However, the levels of Cx46 and Cx50 were similar in homogenates of 1-month-old mice of the 129/C57BL6 and the C3H backgrounds (Supplementary Figure S1), suggesting that there are no intrinsic differences in the levels of Cx46 or Cx50 between the mouse lines used in this study.

### 2.3. Alkaline Phosphatase Treatment

Lenses from 7-day-old Cx50D47A and Cx46fs380 mice were dissected in PBS containing 1 tablet/7 mL cOmplete EDTA-free protease inhibitor cocktail (Roche Applied Science, Indianapolis, IN, USA) and 2 mM PMSF. Lenses were homogenized in the dissection solution followed by sonication. Homogenates were stored at −80 °C until use. Aliquots containing 80 µg of total proteins (to evaluate Cx46) or 20 µg total proteins (to evaluate Cx50) were incubated with or without calf intestinal alkaline phosphatase (M182A; Promega, Madison, WI, USA) overnight at 37 °C in 50 mM Tris-HCl (pH 9.3 at 25 °C), 1 mM MgCl_2_, 0.1 mM ZnCl_2_, and 1 mM spermidine. Then, alkaline phosphatase-treated and untreated samples were subjected to immunoblotting as described above.

### 2.4. Curve Fitting for The Decrease in Connexin Levels

The average densitometric values of the relative density of the immunoreactive bands obtained in three independent sets of experiments were fit to a single exponential of the form: y = C*e^−k*t^, where “C” is the estimated initial densitometric value at day 0, “k” is the rate of decay, and “t” is time (in days) using GraphPad Prism version 8.0.1 for Windows (GraphPad Software, San Diego, CA, USA). To model the degradation of the co-expressed wild-type connexin by its “mixing” with the mutant connexin, we assumed that they could mix in different proportions (containing between 10 and 90% of each connexin). We calculated the values from these two equations for each of the time points, multiplied by different proportions (between 0.1:0.9 and 0.9:0.1), and generated curve fits after adding the values calculated in this manner.

## 3. Results

### 3.1. Levels and Electrophoretic Mobility Patterns of Lens Fiber Connexins Change with Age in Lenses of Wild-Type and Cx46fs380 Mice

We used immunoblotting to determine the electrophoretic migration and abundance of both Cx46 and Cx50 in homogenates prepared from the lenses of homozygous Cx46fs380 mice and their wild-type counterparts at different ages between 1 and 21 days after birth.

**Figure 1:**
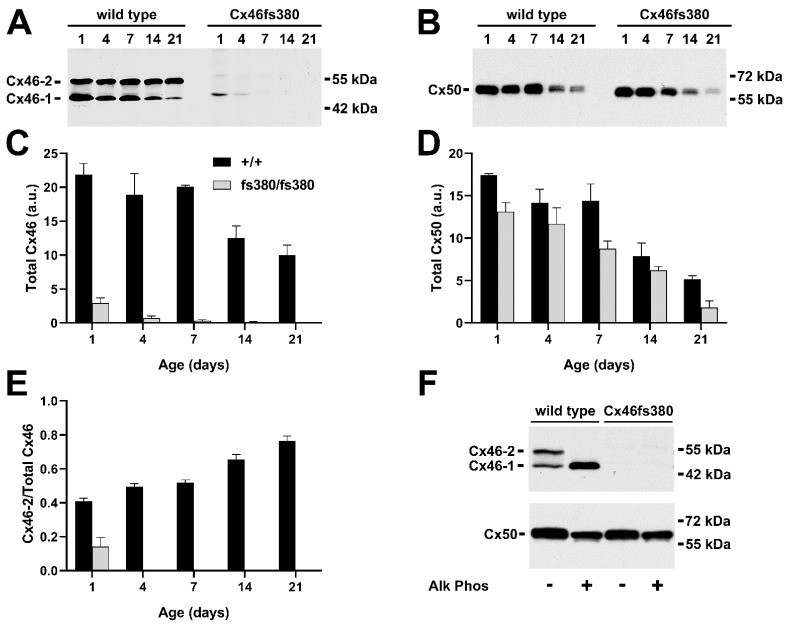
Immunoblot detection and quantification of Cx46 and Cx50 at various post-natal ages in wild-type and Cx46fs380 mice. (**A**,**B**) Immunoblots of Cx46 (**A**) and Cx50 (**B**) in lens homogenates from 1-, 4-, 7-, 14-, and 21-day-old wild-type and homozygous Cx46fs380 mice (129/C57BL6 background). The two major Cx46 bands are indicated as Cx46-1 and Cx46-2. The migration positions of the molecular mass markers are indicated on the right. (**C**,**D**) Graphs show the mean (bar) + SEM of the densitometric values of the immunoreactive Cx46 bands (**C**) and Cx50 bands (**D**) obtained from three independent experiments expressed in arbitrary units (a.u.). (**E**) Graph shows the mean (bar) + SEM of the ratio of Cx46-2 to total Cx46 from densitometric analysis of three independent experiments. (**F**) Immunoblot detection of Cx46 (upper panel) and Cx50 (lower panel) in homogenates from the lenses of 7-day-old wild-type or homozygous Cx46fs380 mice after incubation in the absence or presence of alkaline phosphatase (Alk Phos). The two major Cx46 bands are indicated as Cx46-1 and Cx46-2. The migration positions of the molecular mass markers are indicated on the right.

In the lenses of wild-type mice (mixed 129/C57BL6 background), Cx46 was detected throughout the time course as 2 prominent bands of 46 and 52 kDa (labeled Cx46-1 and Cx46-2, respectively, in Figure 1A). However, the relative proportion of Cx46 contained in the slower migrating band (Cx46-2) became more prominent in samples obtained from older mice. By 21 days, Cx46-2 accounted for 77% of the total Cx46 (Figure 1E).

Cx50 was detected as a broad band of 60–65 kDa between post-natal days 1 and 7 (Figure 1B). At 14 and 21 days, Cx50 appeared as a doublet of bands that migrated similarly to the slowest electrophoretic mobility Cx50 bands detected at earlier ages. Compared to the abundance at post-natal day 1, the total abundance of Cx46 decreased by 54.3%, and the total abundance of Cx50 decreased by 70.1% over the first 21 days after birth (Figure 1C,D). Since the gels were normalized by equal protein loading, some of this decrease may represent the growth of the lens and an increase in the abundance of soluble proteins (crystallins) such that they represented a greater proportion of the lens proteins relative to the connexins.

In lenses from homozygous Cx46fs380 mice, the abundance of Cx46 was much less than that in wild-type mice, even at 1 day of age (13.5%), and the levels became almost undetectable after post-natal day 4 (Figure 1A,C). At days 1 and 4, Cx46 was predominantly composed of a band with an estimated M_r_ of 48 kDa, which closely matches the predicted molecular mass of the Cx46fs380 mutant protein (49 kDa). A small proportion of Cx46 was detected as a slower migrating band (~55 kDa) at day 1 (14%), but it was not detectable at later ages (Figure 1A,E). In lenses from Cx46fs380 mice, the Cx50 immunoreactive bands appeared generally similar to those in the wild-type mice (Figure 1B); however, the total abundance of Cx50 decreased more severely with age in the mutant mice than in the wild-types (Figure 1D, *p* < 0.05 at day 21). No Cx46 or Cx50 bands were detected at lower M_r_, implying that these samples did not contain cleaved products of Cx46 or Cx50.

### 3.2. Phosphorylation of Cx46 and Cx50 in Lenses of Wild-Type and Cx46fs380 Mice

Since both Cx46 and Cx50 are phosphoproteins, we tested whether the different electrophoretic forms of these proteins in wild-type and Cx46fs380 lenses represented posttranslational modification by phosphorylation. We treated homogenates prepared from 7-day-old lenses with alkaline phosphatase and then detected Cx46 and Cx50 by immunoblotting (Figure 1F). This treatment removed the slower mobility form (Cx46-2) detected in untreated wild-type samples and greatly increased the abundance of the faster electrophoretic form (Cx46-1). However, not all of the alkaline phosphatase treated Cx46 migrated identically to Cx46-1; some immunoreactive material migrated a little more slowly (Figure 1F). This observation suggested that Cx46-2 represented phosphorylated Cx46 forms that contained alkaline phosphatase sensitive phosphorylation sites, and that the progressive relative increase in this band in wild-type lenses represented an increasing proportion of phosphorylated Cx46 with age. No Cx46 bands were detected in homogenates of Cx46fs380 lenses in either untreated or alkaline phosphatase treated samples (Figure 1F). For Cx50, alkaline phosphatase treatment caused a small shift in the electrophoretic mobility of immunoreactive Cx50 material in homogenates of both wild-type and homozygous Cx46fs380 lenses, suggesting that both genotypes contained some alkaline phosphatase-sensitive phosphorylated Cx50.

### 3.3. Exponential Decay of Cx46 and Cx50 Levels in Lenses of Wild-Type and Cx46fs380 Mice

**Figure 2:**
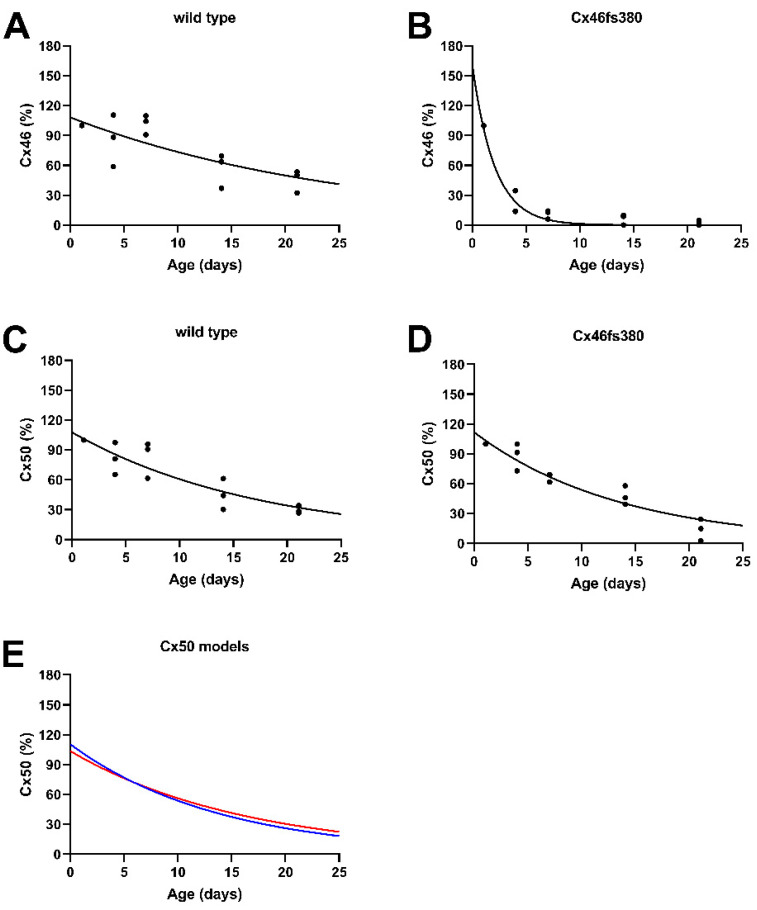
The age-dependent decreases in total levels of Cx46 and Cx50 in the lenses of wild-type and homozygous Cx46fs380 mice could be fit to single exponential curves. (**A**–**D**) The data from the three independent sets of immunoblots of connexin levels shown in Figure 1C,D were fit to single exponential decay curves of the form y = C*e^−k*t^. (**A**,**B**) The data for Cx46 levels were best fit by y = 108.216*e^−0.0387*t^ (r^2^ = 0.854) in wild-type lenses (**A**), and by y = 161.174*e^−0.4807*t^ (r^2^ = 0.997) in Cx46fs380 lenses (**B**). (**C**,**D**) The data for Cx50 levels in wild-type lenses (**C**) were best fit by y = 107.677*e^−0.0574*t^ (r^2^ = 0.9653), and in homozygous Cx46fs380 lenses (**D**), they were best fit by y = 111.807*e^−0.073*t^ (r^2^ = 0.9538). (**E**) The impact of co-oligomerization of wild-type Cx50 with Cx46fs380 upon Cx50 decay in the homozygous Cx46fs380 lenses was modeled by adding different proportions of the curves from panels C and B. The best fit curve was generated using values corresponding to 90% wild-type Cx50 and 10% mutant Cx46, yielding the curve indicated by the red line (y = 110.45*e^−0.07*t^). It is a rather close match to the curve fit to the actual Cx50 data shown in panel D (blue line).

To further analyze the reductions in connexin levels in the wild-type and homozygous Cx46fs380 lenses, we quantified the data from three independent immunoblot experiments and normalized the densities to the value at post-natal day 1 for the corresponding experiment. Then, the data were fit to single exponential decay curves of the form y = C*e^−k*t^ (Figure 2). This analysis showed that in the wild-type animals, the abundances of both Cx46 and Cx50 declined gradually with decay constants (k) of 0.0387 day^−1^ for Cx46 (Figure 2A) and 0.0574 day^−1^ for Cx50 (Figure 2C). As expected, the decay of Cx46 was much faster in the homozygous Cx46fs380 mutant lenses, with a k = 0.4807 day^−1^ (Figure 2B). The decay of Cx50 was also accelerated in the mutant lenses, with a k = 0.073 day^−1^ (Figure 2D).

We hypothesized that the increased disappearance of Cx50 (a wild-type connexin) in the lenses of homozygous Cx46fs380 mice might result from formation of mixed oligomers between Cx50 and the mutant Cx46 destined for degradation. To test this, we modeled the decay of Cx50 in the mutant lenses by adding different proportions of the decay curves for Cx50 in wild-type lenses (Figure 2C) and for Cx46 in the mutant lenses (Figure 2B). We obtained a rather close fit to the actual Cx50 data (presented in Figure 2D) when we used a mixing model containing 90% wild-type Cx50 and 10% mutant Cx46 with a k = 0.061 day^−1^ (Figure 2E). (Compare the red curve representing the values predicted by the model and the blue curve representing the experimental data in Figure 2E).

### 3.4. Levels and Electrophoretic Mobility Patterns of Lens Fiber Connexins Change with Age in Lenses of Wild-Type and Cx50D47A Mice

We also used immunoblotting to determine the abundance and electrophoretic migration of Cx46 and Cx50 in lens homogenates from homozygous Cx50D47A mice and their wild-type counterparts at different ages between 1 and 21 days after birth.

**Figure 3:**
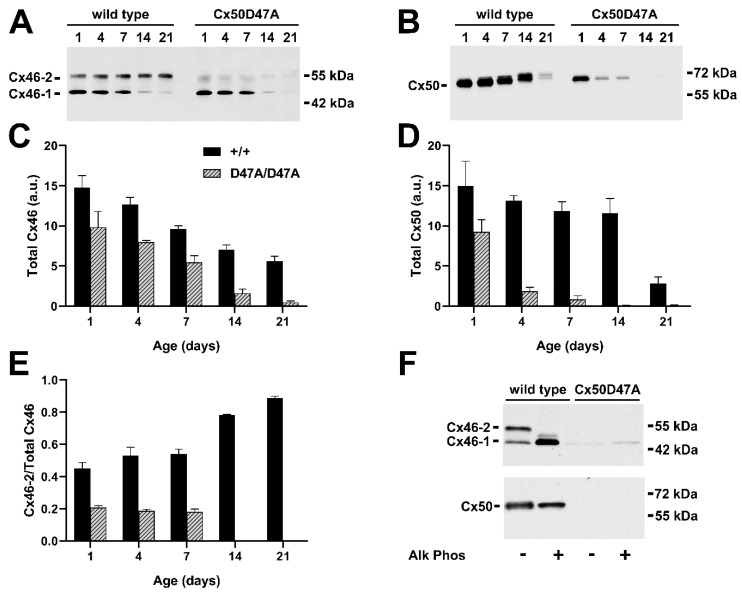
Immunoblot detection and quantification of Cx46 and Cx50 at various post-natal ages in wild-type and Cx50D47A mice. (**A**,**B**) Immunoblots of Cx46 (**A**) and Cx50 (**B**) in lens homogenates from 1-, 4-, 7-, 14-, and 21-day-old wild-type and homozygous Cx50D47A mice (C3H background). The two major Cx46 bands are indicated as Cx46-1 and Cx46-2. The migration positions of the molecular mass markers are indicated on the right. (**C**,**D**) Graphs show the mean (bar) + SEM of the densitometric values of the immunoreactive Cx46 bands (**C**) and Cx50 bands (**D**) obtained from three independent experiments expressed in arbitrary units (a.u.). (**E**) Graph shows the mean (bar) + SEM of the ratio of Cx46-2 to total Cx46 from densitometric analysis of three independent experiments. (**F**) Immunoblot detection of Cx46 (upper panel) and Cx50 (lower panel) in homogenates from the lenses of 7-day-old wild-type or homozygous Cx50D47A mice after incubation in the absence or presence of alkaline phosphatase (Alk Phos). The two major Cx46 bands are indicated as Cx46-1 and Cx46-2. The migration positions of the molecular mass markers are indicated on the right.

In the lenses of wild-type mice (C3H background), the changes in abundances and in the different electrophoretic mobility forms of Cx46 and Cx50 were generally similar to our observations in the wild-type mouse lenses on the mixed 129/C57BL6 background. Cx46 was detected as two prominent bands of about 46 and 52 kDa (Cx46-1 and Cx46-2 in Figure 3A). The relative proportion of Cx46-2 increased in samples obtained from older mice; by 21 days, Cx46-2 accounted for 89% of the total Cx46 (Figure 3A,E). At post-natal day 1, Cx50 was detected as a broad band of 60-65 kDa. Over subsequent days, the electrophoretic mobility of immunoreactive Cx50 decreased and multiple separate bands were resolved in some samples (Figure 3B). Over the first 21 days of post-natal life, the total abundance of Cx46 decreased by 62%, and the total abundance of Cx50 decreased by 80% with respect to the levels detected at post-natal day 1 (Figure 3C,D).

In lenses from homozygous Cx50D47A mice, Cx46 was predominantly detected at the electrophoretic mobility of Cx46-1, representing ~80% of total Cx46 at all ages where substantial amounts of Cx46 were detectable (Figure 3A,E). Additionally, the total abundance of Cx46 decreased much faster in the homozygous Cx50D47A mice than in the wild-type counterparts (Figure 3C). In contrast to the changes in electrophoretic mobility of Cx50 in wild-type lenses, the mobility of Cx50 bands did not appear different at any of the ages studied in the homozygous Cx50D47A lenses (Figure 3B). The total abundance of Cx50 decreased much more rapidly in the homozygous than in the wild-type animals; it was nearly undetectable at 14 and 21 post-natal days in the Cx50D47A mutant mice (Figure 3B,D). We did not detect Cx46 or Cx50 bands at lower M_r_, suggesting the absence of cleaved forms of Cx46 or Cx50 in these samples.

### 3.5. Phosphorylation of Cx46 and Cx50 in Lenses of Wild-Type and Cx50D47A Mice

The alkaline phosphatase treatment of homogenates prepared from the lenses of wild-type mice of the C3H background produced results that were generally similar to those obtained in lenses of wild-type mice of the mixed 129/C57BL6 background (Figure 3F). The slower migrating band of Cx46 (Cx46-2) present in the untreated sample of wild-type lenses collapsed into the faster migrating band (Cx46-1), consistent with Cx46-2 representing phosphorylated forms (Figure 3F). In these experiments, we clearly detected a Cx46 band of intermediate mobility (just above Cx46-1) that may represent Cx46 with a different post-translational modification or modified by phosphate that was not removed by the alkaline phosphatase treatment (Figure 3F). The breadth of the Cx50 band was somewhat reduced by the treatment with alkaline phosphatase (Figure 3F). In lens samples from homozygous Cx50D47A mice, the major Cx46 band (Cx46-1) was weakly detected, but unaffected in mobility or intensity by alkaline phosphatase treatment. Comparable gel loading and exposure yielded no detectable Cx50 bands in the untreated or alkaline phosphatase-treated sample from a Cx50D47A lens homogenate.

Taken together with the images shown in Figure 3A,B, these results imply that phosphorylation of Cx46 (and likely Cx50) is greatly reduced in the lenses of Cx50D47A mutant mice as compared to wild-type animals.

### 3.6. Exponential Decay of Cx46 and Cx50 Levels in Lenses of Wild-Type and Cx50D47A Mice

**Figure 4:**
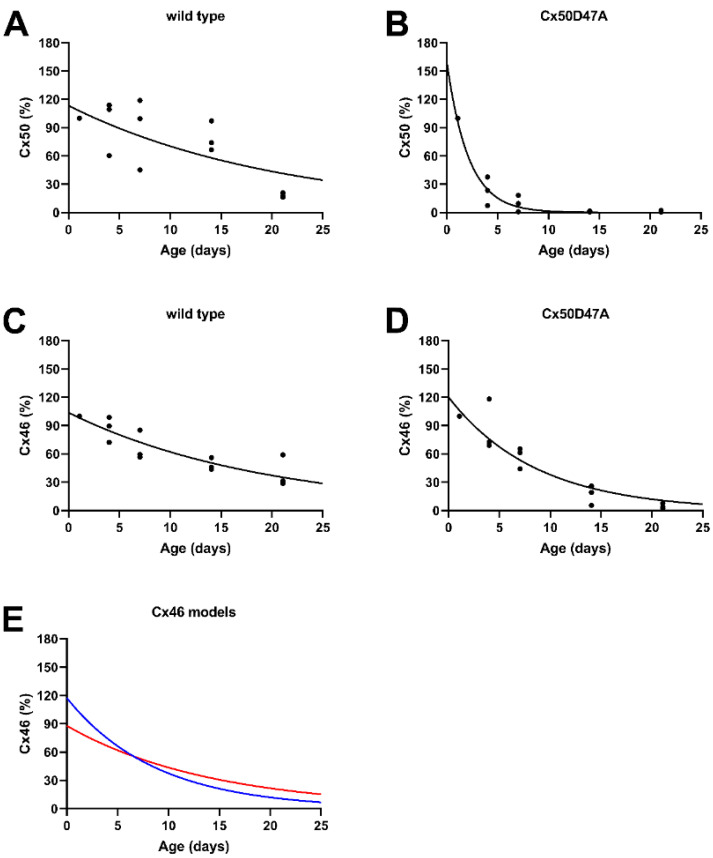
The data regarding the age-dependent decreases in total levels of Cx46 and Cx50 in the lenses of wild-type and homozygous Cx50D47A mice could be fit to single exponential curves. (**A**–**D**) The data from the three independent sets of immunoblots of connexin levels shown in Figure 3C,D were fit to single exponential decay curves of the form y = C*e^−k*t^. (**A**) For Cx50 levels in wild-type (C3H background) mice, the data were best fit by y = 113*e^−0.0477*t^ (r^2^ = 0.7333). (**B**) For Cx50 levels in Cx50D47A mice, the data were best fit by y = 159.095*e^−0.4665*t^ (r^2^ = 0.9944). (**C**) For Cx46 levels in wild-type mice (C3H background), the data were best fit by y = 103.7676*e^−0.0513*t^ (r^2^ = 0.9516). (**D**) For Cx46 levels in Cx50D47A mice, the data were best fit by y = 120.179*e^−0.1151*t^ (r^2^ = 0.99418). (**E**) The impact of co-oligomerization between wild-type Cx46 and Cx50D47A upon Cx46 decay in the Cx50D47A mice was modeled by adding different proportions of the values from the curves shown in panels C and B. This approach did not yield a very satisfactory match. The example shown as a red line represents a curve generated using 60% wild-type Cx46 (**C**) and 40% mutant Cx50 (**B**). The curve fit to the actual Cx46 data (from panel **D**) is shown as a blue line (which could also be generated by progressively reducing the relative proportion of wild-type Cx46 from 86% at day 1 to 30% at day 21).

To test whether the reductions in connexin levels in the lenses of wild-type and homozygous Cx50D47A mice presented in Figure 3 also followed a single exponential, we normalized the densities of the immunoreactive bands obtained from three independent experiments to the value obtained at post-natal day 1 for the corresponding experiment and fitted the values to a curve of the form y = C*e^−k*t^. For the wild-type mice in the C3H background, this analysis revealed that the abundances of both Cx46 and Cx50 declined gradually with decay constants of 0.0477 day^−1^ for Cx50 (Figure 4A) and 0.0513 day^−1^ for Cx46 (Figure 4C). These decay constants were similar to those obtained for lenses of wild-type mice from the mixed 129/C57BL6 background. In comparison, in the homozygous Cx50D47A mutants, the decay of Cx50 was much faster with a k = 0.4665 day^−1^ (Figure 4B). The decay of Cx46 was also accelerated in the mutant lenses with a k = 0.1151 day^−1^ (Figure 4D).

We also assessed whether the degradation of wild-type Cx46 in the Cx50D47A mice could be explained by mixing of wild-type Cx46 with the mutant Cx50 by adding different proportions of the curves that had been fit to the wild-type Cx46 and the mutant Cx50 data. However, we were unable to identify a combination of the two curves that fit the data very closely. Figure 4E shows the curve obtained with the combination that approximated better to the data (60% Cx46 and 40% mutant Cx50, illustrated with a red line, k = 0.072), but it still has a poor correspondence to the curve fit to the actual Cx46 data (blue line). A better fit for the Cx46 data was obtained only if the proportion of mutant and wild-type connexins was varied at each time point; this required changing the proportion of mixing from 86% wild-type Cx46 and 14% mutant Cx50D47A at post-natal day 1 to 30% wild-type Cx46 and 70% mutant Cx50 at post-natal day 21 (matching the blue line in Figure 4E).

## 4. Discussion

In the current study, the total levels of both Cx46 and Cx50 decreased between 1 and 21 days after birth in wild-type animals. Because the gels were loaded with equal amounts of lens protein in each lane, these data show a decrease in the relative abundance of the connexins compared to total lens proteins (not necessarily a change in the absolute connexin abundance). This relative decrease likely results from the significant increase in abundance of other lens proteins (mainly crystalllins) at these ages, associated with the lens growth that occurs over this period. Although unlikely, we cannot discard the possibility that some of the connexins may have undergone full degradation as the lens continued to grow over the time frame of our studies.

The change in the electrophoretic banding pattern of the lens fiber cell connexins in wild-type lenses with increasing age likely represents post-translational “maturation” of the proteins. This interpretation is substantiated by the results of the alkaline phosphatase treatment experiments, which show that most of the immunoreactive connexin collapsed into the band with the fastest electrophoretic mobility. In addition, these experiments indicate that the change in the electrophoretic mobility primarily represents the phosphorylation of the proteins. Both Cx46 and Cx50 are known to be phosphoproteins [6,19,20,21,22,23,24]. Previous immunoblotting experiments using ovine, bovine, and rat samples have shown that alkaline phosphatase treatment modifies the electrophoretic mobility of immunoreactive Cx46 and Cx50 [6,19,20,21,22]. In rat lenses, Cx46 is mainly detected as two bands of 46 and 54 kDa; both forms are present at embryonic ages, but the slower (phosphorylated) form predominates in young adults [21]. Our alkaline phosphatase experiments also showed the presence of immunoreactive Cx46 with a modification that was not sensitive to alkaline phosphatase treatment. This may not be surprising since other modifications (including acetylation, nitrosylation, and carbamylation) have been detected in Cx46 (reviewed in Retamal and Altenberg [25]). In Cx43, some phosphates are removed by acid phosphatase [26].

Our previous studies in transfected cells have elucidated cell biological aspects of the defects conferred by these connexin mutations. The Cx50D47A mutant is largely retained in the biosynthetic pathway [27], and, therefore, it forms few (if any) gap junctions at the plasma membrane. Immunofluorescence performed using flatmounts of the lens epithelium from Cx50D47A mice confirms that most of the immunoreactive Cx50 localizes in the cytoplasm [9]. For Cx46fs380, the abnormal cytoplasmic tail created by the frameshift mutation generates a retrieval/retention signal in the mutant protein [28], limiting the formation of gap junctions. In the current study, we detected a small amount of Cx46fs380 protein by immunoblotting at post-natal day 1 (Figure 1A). Moreover, some Cx46fs380 was detected at a slower mobility (~55 kDa), consistent with it being phosphorylated like the wild-type protein. This observation is consistent with our previous observation of a near absence of Cx46 gap junctional plaques in 1-month-old mice that were homozygous for Cx46fs380 [8]. In the Cx50D47A lenses, Cx46 also seemed to be post-translationally modified as some of it migrated with an M_r_ of ~55 kDa.

The faster decrease in levels of the mutant connexin in homozygous Cx46fs380 and Cx50D47A lenses compared with the decrease in Cx46 and Cx50 in the corresponding wild-type lenses most likely represents the increased degradation of the mutant proteins. Since both of the mutants are retained in components of the biosynthetic pathway, it is likely that they are degraded as the cells differentiate and degrade their organelles to become mature lens fiber cells.

In both mutant mouse lines, the expression of a mutant lens fiber cell connexin affected the co-expressed wild-type connexin. However, some of the effects (and likely the mechanisms underlying them) were different.

In Cx46fs380 mice, the electrophoretic mobility of the different Cx50 forms appeared largely unaffected by the expression of the Cx46 mutant (Figure 1B). In contrast, in Cx50D47A lenses only a very small fraction of Cx46 was detected as the Cx46-2 form (Figure 3A), implying that expression of the mutant Cx50 in the lens had major effects on the post-translational modification of Cx46.

Cx46 and Cx50 can form mixed (heteromeric) channels, as shown by their co-immunoprecipitation from sheep lens membranes [5] and by their formation of channels with properties that differ from those of the homomeric Cx46 or Cx50 channels when co-expressed in *Xenopus* oocytes [29] or in transfected cells [30]. However, the true extent of mixing between wild-type Cx50 and wild-type Cx46 in the lens (as well as their true absolute amounts) are not known. The accelerated decay of wild-type Cx50 in homozygous Cx46fs380 lenses compared to wild-type lenses can be explained by a relatively small amount (10%) of mixing (co-oligomerization) between wild-type Cx50 and the mutant Cx46fs380 (Figure 2E). Since Cx46fs380 has a retention/retrieval signal, it is likely that some mixed oligomers (containing Cx46fs380 and wild-type Cx50) are retained in the biosynthetic pathway and subsequently degraded. The relatively estimated small amount of co-oligomerization between these proteins may reflect the differential abundances of the proteins in epithelial and fiber cells. Cx46 is almost exclusively expressed in fiber cells [8,31,32]. In contrast, there is substantial expression of Cx50 in lens epithelial cells [32], especially at early postnatal ages when Cx50-mediated coupling accounts for 63% of the coupling in epithelial cells [33]. Therefore, epithelial Cx50 must contribute significantly to the total Cx50 level at early ages.

In contrast, Cx50D47A has major effects on both the maturation/modification of the Cx46 protein and its stability. In these animals, Cx46 shows very little shift between Cx46-1 and Cx46-2 forms. The faster decay of wild-type Cx46 in Cx50D47A lenses as compared to wild-type lenses cannot be explained by the mixing of a constant amount of the wild-type Cx46 with the mutant Cx50. Although the data could be fit by assuming an age-dependent increase in the proportion of mutant Cx50 co-oligomerizing with wild-type Cx46, we consider this interpretation extremely unlikely because the levels of the mutant Cx50 approached zero at the later time points. While co-oligomerization between wild-type Cx46 and Cx50D47A may contribute to the decrease in the wild-type connexin, it is likely that the impaired differentiation [9] and induction of endoplasmic reticulum stress [15] in these lenses have more significant roles. Either of these processes might have affected levels of Cx46 and its post-translational modifications by altering protein synthesis and degradation pathways or kinase activities. In addition, the phosphorylation state of the connexins might have affected hetero-oligomerization.

These findings might have implications for other cell types because multiple connexins are co-expressed in many different kinds of cells. Cx40, Cx43, and Cx45 are co-expressed in cardiac myocytes [34,35]. Cx32 and Cx26 are both present in hepatocytes [36], and levels of Cx26 are reduced in Cx32 knock-out mice [37]. Many different connexins (including Cx26, Cx30, Cx30.3, Cx31.1, Cx31, and Cx43) are expressed in the epidermal cells of the skin (reviewed in Scott et al. [38]), and some Cx26 mutants have been shown to affect co-expressed Cx43 even though Cx26 and Cx43 do not normally co-oligomerize [39]. Our data suggest that a mutant of one of these connexins might affect the phosphorylation and stability of the other co-expressed connexins through different mechanisms, either directly (through co-oligomerization followed by degradation) or indirectly, by stimulating major cellular pathways. The consequent alterations in connexin abundance and its post-translational modifications (which often result in changes in channel abundance and/or function) might alter the extent of intercellular communication in these cells.

## Data Availability

Not applicable.

## Supplementary Materials

The following supporting information can be downloaded at: https://www.mdpi.com/article/10.3390/cells11182786/s1, Figure S1: Wild-type lenses of two different genetic backgrounds have comparable levels of the lens fiber cell connexins.
