# Supplementary material for: Levels and Modifications of Both Lens Fiber Cell Connexins Are Affected in Connexin Mutant Mice
Source: Cells. 2022 Sep 7;11(18):2786. doi: 10.3390/cells11182786 (PMC9496683; doi:10.3390/cells11182786)
Supplement: Supplementary file 1 [file cells-11-02786-s001.zip › cells-1878692-supplementary.pdf]

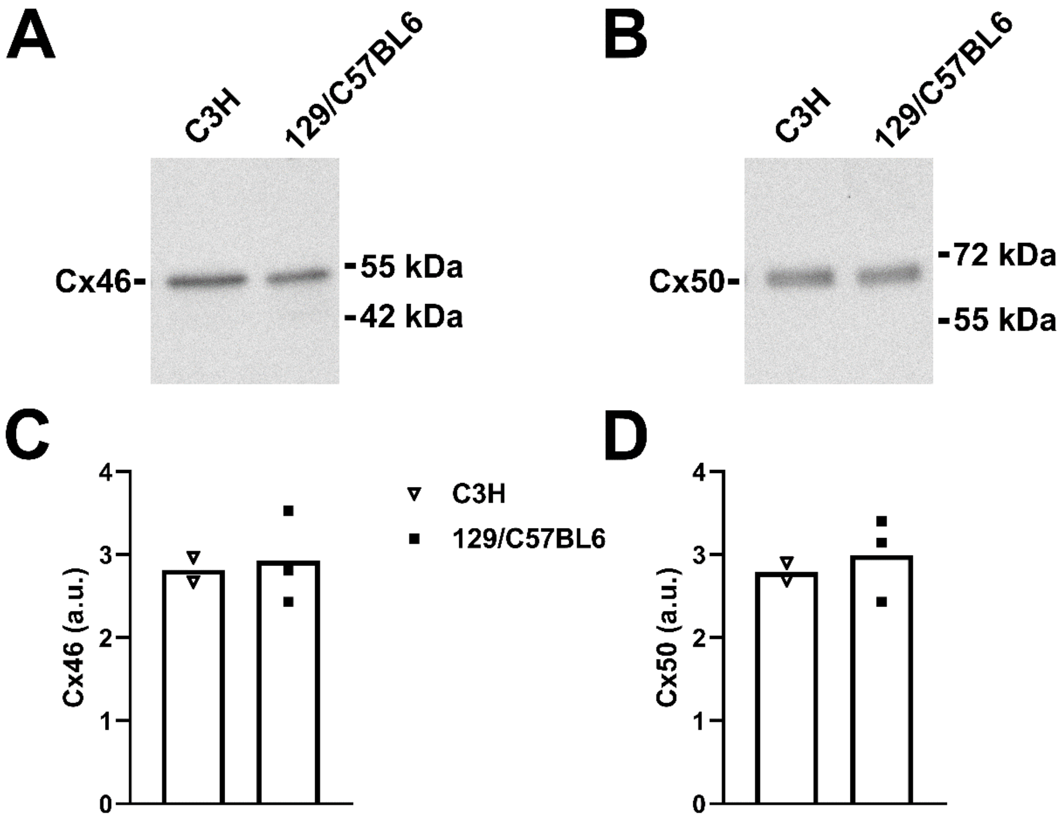

Supplementary Figure S1. Wild type lenses of two different genetic backgrounds have comparable levels of the lens fiber cell connexins. (A, B) Immunoblots of Cx46 (A) and Cx50 (B) in lens homogenates from 1-month old wild type mice of the C3H and 129/C57BL6 genetic backgrounds. The migration positions of the molecular mass markers are indicated on the right. (C, D) Graphs show the densitometric values of the immunoreactive Cx46 (C) and Cx50 (D) bands.
